# Exogenous avian leukosis virus-induced activation of the ERK/AP1 pathway is required for virus replication and correlates with virus-induced tumorigenesis

**DOI:** 10.1038/srep19226

**Published:** 2016-01-12

**Authors:** Manman Dai, Min Feng, Yu Ye, Xiaochan Wu, Di Liu, Ming Liao, Weisheng Cao

**Affiliations:** 1 College of Veterinary Medicine, South China Agricultural University, Guangzhou, 510642, People’s Republic of China; 2 College of Animal Science, South China Agricultural University, Guangzhou, 510642, People’s Republic of China; 3 Key Laboratory of Veterinary Vaccine Innovation of the Ministry of Agriculture; 4 South China Collaborative innovation Center for Prevention and Control of poultry Infectious diseases and Safety of Poultry Products.

## Abstract

A proteomics approach was used to reveal the up-regulated proteins involved in the targeted mitogen-activated protein kinase (MAPK) signal transduction pathway in DF-1 cells after ALV subgroup J (ALV-J) infection. Next, we found that ALV-J CHN06 strain infection of DF-1 cells correlated with extracellular signal-regulated kinase 2 (ERK2) activation, which was mainly induced within 15 min, a very early stage of infection, and at a late infection stage, from 108 h to 132 h post-infection. Infection with other ALV subgroup (A/B) strains also triggered ERK/MAPK activation. Moreover, when activating ERK2, ALV subgroups A, B and J simultaneously induced the phosphorylation of c-Jun, an AP1 family member and p38 activation but had no obvious effect on JNK activation at either 15 min or 120 h. Interestingly, only PD98059 inhibited the ALV-induced c-Jun phosphorylation while SP600125 or SB203580 had no influence on c-Jun activation. Furthermore, the viral gp85 and gag proteins were found to contribute to ERK2/AP1 activation. Additionally, the specific ERK inhibitor, PD980509, significantly suppressed ALV replication, as evidenced by extremely low levels of ALV promoter activity and ALV-J protein expression. *In vivo* analysis of ERK2 activation in tumor cells derived from ALV-J-infected chicken demonstrated a strong correlation between ERK/MAPK activation and virus-associated tumorigenesis.

Avian leukosis viruses (ALV), an oncogenic retrovirus, mainly induce neoplastic diseases and other reproduction problems by both vertical and horizontal transmission infection. Based on the identity of viral envelope glycoproteins, ALV is divided into subgroups A, B, C, D, E, J and the recently identified subgroup K^1^,^2^. So far, most of isolated and identified ALV in China pertain to subgroups A, B and J inducing disease in the commercial meat and egg type chickens as well as in the Chinese local breeds^3^,^4^,^5^,^6^. ALV can cause significant economic losses as a result of tumor-induced mortality, serious immunosuppression and reduction of weight gain, egg production and breeding potential. Widespread ALV-J can induce various tumors in chickens, especially myeloid leukosis and hemangioma. Our previous study demonstrated that MYC, TERT, and ZIC1genes were identified as the common insertion sites in ALV-J induced myeloid leukosis, which might be a putative “driver” for the activation of the oncogene^7^. However, the cellular factors involved in exogenous ALV infection and oncogenesis, especially signal transduction pathways, are yet to be revealed.

Isobaric tags for relative and absolute quantification (iTRAQ) is a stable isotope method for protein measurement by using mass spectrometry^8^. In the current study, we applied iTRAQ followed by liquid chromatography/tandem mass spectrometry (LC-MS/MS) to compare the protein profile of DF-1 cell line with and without ALV-J infection. Based on the proteome data and Western blot confirmation, we inferred that the up-regulation of mitogen-activated protein kinase1 (MAPK) which is closely related to ERK/MAPK signaling pathway may play a role in ALV-J propagation, cell proliferation and neoplasm formation.

MAPK cascades are composed of four prototype members, the extracellular signal-regulated kinase (ERK) 1/2, p38, the Jun-N-terminal kinase (JNK/SAPK) and ERK5, which are key signaling pathways in the control of cell proliferation, differentiation, apoptosis and immune responses^9^,^10^,^11^,^12^. Aberrant regulation of MAPK cascades incurs neoplasm and other diseases. In particular, the ERK/MAPK pathway has been the subject of intense study on treatment of cancer^12^. ERK1/2 is downstream of the growth-factor-stimulated Raf/MEK/ERK signaling cascade, while p38 and JNK/SAPK are activated by apoptosis signal-regulating kinase 1^13^. Activator protein 1 (AP1) regulates a wide range of cellular processes, including cell proliferation, death, survival and differentiation, and is induced by a plethora of physiological stimuli and environmental insults. AP-1, as the downstream of MAPK cascade, can be activated by any or all three main of the MAPK pathways through phosphorylation of distinct substrates^14^. The AP-1 complex is consisted of heterodimers of Jun (c-Jun, JunB and JunD) and Fos (c-Fos, Fos B, Fra1 and Fra2) family members, or Jun homodimers. In particular, c-Jun has been implicated in events leading to tumor development^15^,^16^,^17^,^18^.

Maintenance of the oncogenic phenotype depends on the accumulation of genetic changes, as well as epigenetic events such as the induction of specific promotion-relevant effector genes. Certain target genes of the AP-1 transcription factor complex are thought to mediate in neoplastic transformation, although the identity of these genes remains largely unknown^19^. Vascular endothelial growth factor (VEGF) is a highly specific mitogen for vascular endothelial cells. The expression of VEGF is potentiated in response to hypoxia, by activated oncogenes, and by a variety of cytokines. VEGF induces endothelial cell proliferation, promotes cell migration, and inhibits apoptosis^20^,^21^. Activated oncogenes that are part of the ras/MAP-kinase signal transduction pathway can up-regulate *VEGF* mRNA expression^22^,^23^. Since MAPK/AP-1 signal transduction pathway is found to be associated with oncogenesis, ALV-J-induced tumor in chickens may be associated with the activation of MAPK/AP-1 pathway.

In this study, we investigated the role of MAPK/AP-1 regulation during exogenous ALV propagation *in vitro* and ALV-J oncogenesis *in vivo*. Our findings extend our current understanding of the exogenous ALV infection and oncogenecity mechanisms.

## Results

### MAPK protein is up-regulated in ALV-J-infected DF-1 cells

**Figure 1:**
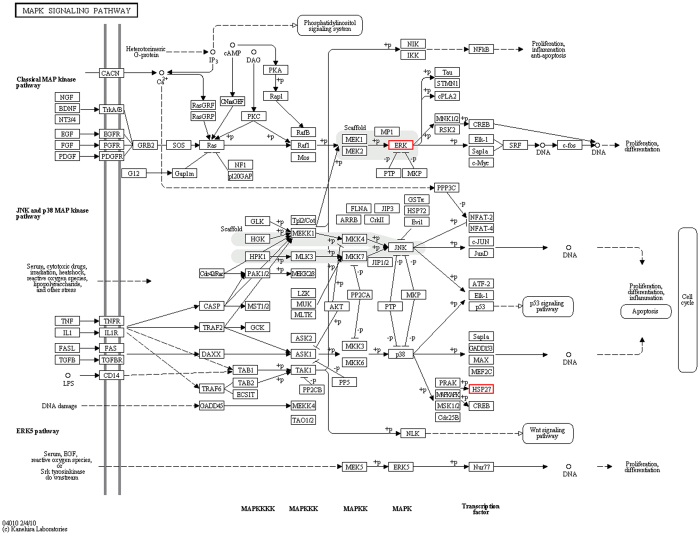
MAPK signaling pathway analysis of proteins that were significantly altered in ALV-J-infected DF-1 cells. Red means up-regulated proteins. The mapped pathway of the increased proteins in ALV-J-infected DF-1 cells was obtained from the KEGG PATHWAY Database (http://www.genome.jp/kegg-bin/show_pathway?map04010)^81^.

**Figure 2:**
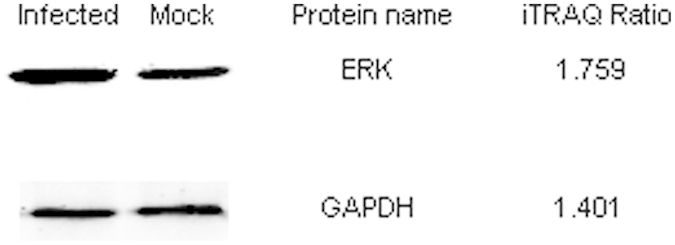
Confirmation of MAPK protein levels by western blotting. DF-1 cells were infected by live viral stocks of ALV-J CHN06 for 60 h at a multiplicity of infection (MOI) of 1. Mock-infected DF-1 cells were used as a negative control. Cell lysates were subjected to western blotting with rabbit anti-p44/42 MAPK and GAPDH antibodies. iTRAQ ratios are shown on the right side.

We compared the differences in the levels of the isolated proteins from ALV-J-infected and mock-infected DF-1 cells by quantitative proteomics. Using a strict cutoff value of 1.5-fold for expressed variation^24^ and using the GO function significant sexual enrichment analysis, we found some differentially expressed proteins involved in the MAPK signaling pathway (Fig. 1). And the up-regulated MAPK protein was further verified by Western blotting (Fig. 2).

### Exogenous ALV infection activates ERK2/AP-1 pathway in DF-1 cells

**Figure 3:**
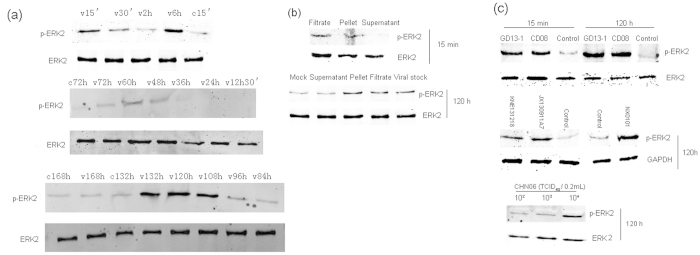
ALV infection activates ERK2 phosphorylation. DF-1 cells were infected by live viral stocks of ALV-J CHN06 (10^4^ TCID_50_ 0.2 ml^−1^), ALV-J NX0101 (10^4^ TCID_50_ 0.2 ml^−1^), ALV-A GD13-1 (10^4^ TCID_50_ 0.2 ml^−1^), ALV-B CD08 (10^4^ TCID_50_ 0.2 ml^−1^) or wild strains of ALV-J KNE131218 (S/P ratio of 1.8) and JX130911A7 (S/P ratio of 2.2). Mock-infected DF-1 cells in various phases were used as a negative control. Cell lysates prepared at the indicated times p.i. were subjected to SDS-PAGE and the amounts of phosphorylated ERK1/2 (p-ERK1/2) and total ERK1/2 were evaluated by western blotting. Chickens solely express the 42-kDa ERK2 isoform. (**a**) The activation profile of ERK2 was explored in CHN06-infected DF-1 cells. (**b**) Viral stocks of CHN06 were filtered and pelleted. The filtrate, pellet and supernatant were used individually to infect DF-1 cells for 15 min and 120 h. (**c**) ERK2 phosphorylation was examined in DF-1 cells infected by ALV-A or B, NX0101 or wild strains of ALV-J, or various virus titers of CHN06, at 15 min or at 120 h p.i. The results are representative of two to three independent experiments.

Given the significant correlation between the ERK/MAPK signaling pathway and tumorigenesis, we initially investigated whether ALV-J strain CHN06 leads to activation of this pathway during the infection process. Chickens solely express the 42-kDa ERK2 isoform, but not ERK1^25^,^26^, which is consistent with our results. In contrast to the mock-treated cell lysate results, CHN06 infection led to a rapid and transient increase in ERK phosphorylation within 15 min p.i., which declined by 30 min p.i., and returned to the basal level by 2 h p.i. ERK phosphorylation increased again at 6 h p.i., subsiding by 12.5 h p.i., and then activation resumed between 108 h and 132 h p.i. (Fig. 3a). These results suggest that ALV-J-induced activation of the MAPK pathway occurred mainly at the early and late infection stages.

To eliminate any secreted factors in the conditioned media associated with the viral stock that could have triggered the production of phosphorylated ERK (p-ERK), viral stocks were filtered and pelleted by centrifugation, and then suspended and inoculated onto DF-1 cells. Figure 3b demonstrates that the viral filtrate and pellet from the viral stocks of CHN06 induced p-ERK at 15 min and 120 h p.i., showing the same activation pattern as that by the viral stocks from the infected cell culture supernatants. No signal was detected in the cellular extraction of DF-1 cells inoculated with supernatant after centrifugation. These results indicate that CHN06 virus particles, but not the secreted factors associated with the viral stock, activated ERK at the stage of cell infection.

To explore whether other ALV subgroups can also activate ERK/MAPK in DF-1 cells, we examined the level of ERK phosphorylation at 15 min and 120 h p.i. with ALV-A strain GD13-1 or ALV-B strain CD08. Similar to the results of ALV-J strain infection, both GD13-1 and CD08 activated ERK phosphorylation as early as 15 min and as late as 120 h after infection of DF-1 cells. Moreover, ALV-J strain NX0101 and two strains of ALV-J isolated from clinical plasma samples, which were numbered KNE131218 and JX130911A7, increased p-ERK expression at 120 h p.i. Furthermore, the expression levels of ERK phosphorylation increased with the ALV-J strain CHN06 virus titer at 120 h p.i. (Fig. 3c). Accordingly, we concluded that there was no significant difference among the exogenous ALV subgroups in activating ERK/MAPK in DF-1 cells, and that ALV infection led to increased ERK phosphorylation, which was virus titer-dependent.

**Figure 4:**
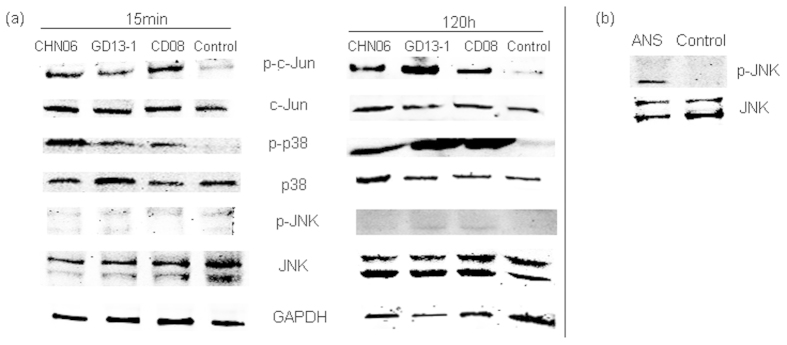
ALV infection significantly activates AP-1 and p-p38, but not JNK phosphorylation, at 15 min and 120 h p.i. DF-1 cells were mock infected or infected by CHN06, GD13-1 or CD08 at 10^4^ TCID_50_ 0.2 ml^−1^. (**a**) After 15 min or 120 h, cell lysates were collected and the amounts of p-c-Jun, c-Jun, p-JNK, total JNK, p-p38, total p38 and GAPDH were evaluated by western blotting. (**b**) DF-1 cells were treated with the JNK activator, Anisomycin (20 μM) for 12 hours according to the instructions, and cell lysates were collected and the amounts of p-JNK, total JNK were evaluated by western blotting. The results are representative of two to three independent experiments. Abbreviations: ANS, Anisomycin.

To investigate whether exogenous ALV infection can also activate the other two MAPK pathways in the same way, we examined the level of p38 and JNK phosphorylation at 15 min and 120 h p.i. with ALV-J strain CHN06, ALV-A strain GD13-1 and ALV-B strain CD08. The results showed that the virus stocks of all exogenous ALV infections could activate p-p38, but had no obvious effect on JNK activation at 15 min and 120 h, which was analogous to the mock-treated cell lysate results (Fig. 4a). And JNK activator, Anisomycin indeed activated p-JNK in DF-1 cells (Fig. 4b), which eliminated the factors of reagents and operations in the experiments. Besides, ALV infection increased the phosphorylation of c-Jun at 15 min and 120 h p.i. (Fig. 4a).

**Figure 5:**
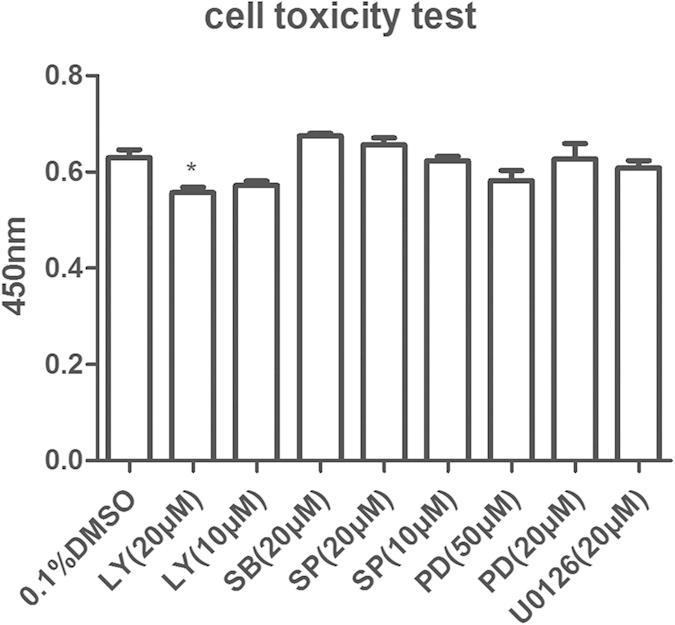
Drug cell toxicity test. DF-1 cells were treated with LY294002 (10–20 μM), SB203580 (20 μM), SP600125 (10–20 μM), PD98059 (20–50 μM), U0126 (20 μM) or DMSO (0.1%, v/v). Cytotoxicity test was performed with Cell Counting Kit-8 dye. Data are representative of two independent experiments, both performed in triplicate. ***p < 0.001. Error bars indicate SEM. Abbreviations: LY, LY294002; SB, SB203580; SP, SP600125; PD, PD98059.

**Figure 6:**
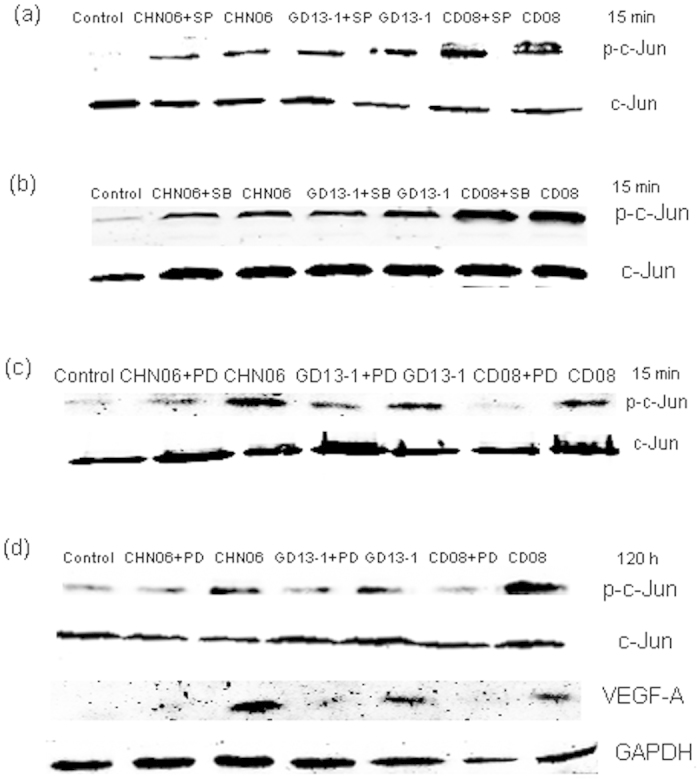
AP-1 is activated by ERK/MAPK pathway. DF-1 cells were preincubated with 20 μM SP600125 (**a**), 20 μM SB203580 (**b**), 20 μM PD98059 (**c**) and 50 μM PD98059(**d**) for 1 h and subsequently infected with CHN06, GD13-1 or CD08 at 10^4^ TCID_50_ 0.2 ml^−1^. The inhibitors were in the cell culture fluid all the time. Cell lysates were collected and the amounts of p-c-Jun, c-Jun, VEGF-A and GAPDH were evaluated by western blotting. The results are representative of two independent experiments.

To explore which one of the three MAPK pathways induced the phosphorylation of c-Jun, we examined the influences of various MAPK pathway inhibitors on c-Jun activation. Additionally, a CCK-8 assay was performed on DF-1 cells treated with different concentrations of inhibitors, to pick the right concentrations without affecting cell viability. Various inhibitors have no cell toxicity except 20 μM LY294002 (Fig. 5). Surprisingly, only PD98059 inhibited the ALV-induced c-Jun phosphorylation while SP600125 or SB203580 had no influence on c-Jun activation at 15 min p.i (Fig. 6a–c). Hence, we deduced that ALV infection activated AP-1 family members mainly through the ERK signaling pathway in DF-1 cells. More importantly, PD98059 simultaneously inhibited the ALV-induced c-Jun phosphorylation and VEGF-A expression at 120 h p.i (Fig. 6d).

**Figure 7:**
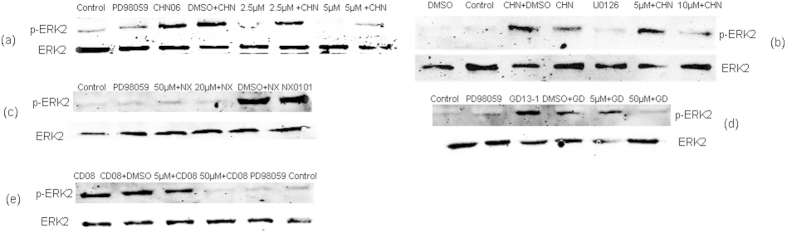
ALV infection activates ERK2 phosphorylation in a MEK-dependent manner. DF-1 cells were preincubated with PD98059 (2.5–50 μM), U0126 (5–10 μM) or DMSO (0.1%, v/v) for 1 h and subsequently infected with CHN06 (**a**,**b**), GD13-1 (**d**) or CD08 (**e**) for 15 min, or with NX0101 (**c**) for 120 h, at 10^4^ TCID_50_ 0.2 ml^−1^. The inhibitors or DMSO were in the cell culture fluid all the time. And the concentration of inhibitor control is the maximum concentration used in each independent experiment. Cell lysates were collected and the amounts of p-ERK2 and total ERK were evaluated by western blotting. The results are representative of two independent experiments. Abbreviations: CHN, CHN06 or HN06; NX, NX0101; GD13-1, GD; CD08, CD.

To determine whether the phosphorylation of ERK in DF-1 cells was MEK1/2-dependent or -independent, the role of MEK1/2 in ERK activation following ALV infection was investigated. As expected, ALV infection increased the phosphorylation of ERK, while PD98059 or U0126 pretreatment inhibited the ALV-induced ERK phosphorylation in a concentration-dependent manner (Fig. 7a–e).

### *Gp85* or *gag* protein alone induces ERK2/AP1 activation

**Figure 8:**
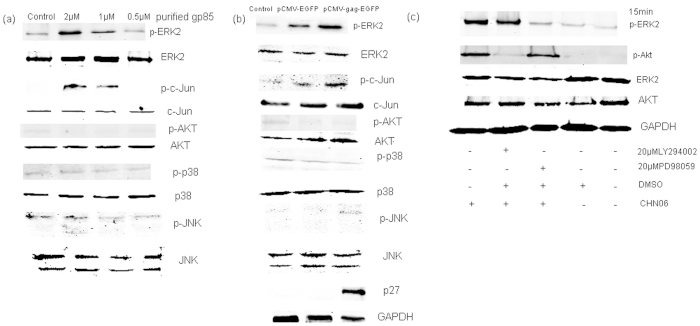
gp85 or gag protein alone induces ERK2/AP1activation. Cell lysates were analyzed by western blot analysis of kinase activation using antibodies specific for the phosphorylated forms of the proteins. (**a**) DF-1 cells were stimulated for 10 min with different concentrations of purified gp85, followed by western blot analysis of phosphorylated proteins and total proteins. (**b**) DF-1 cells were electroporated with pCMV-gag-EGFP or vector. Seventy-two hours later, cell lysates were subjected to western blotting with phosphorylated proteins, total proteinsp27 or GAPDH antibodies. (**c**) DF-1 cells were incubated for 1 h in the presence or absence of the PI3K inhibitor, LY294002 (20 μM), the ERK/MAPK inhibitor, PD98059 (20 μM), or DMSO (0.1%, v/v), and subsequently infected with CHN06 for 15 min at 10^4^ TCID_50_ 0.2 ml^−1^, followed by western blot analysis of ERK and Akt activation. Data shown are representative of two independent experiments

To investigate which viral proteins are involved in ALV activation of the ERK2/AP-1 pathway, we focused on gp85 protein, which is easier to mutate, and gag protein, whose gene is relatively conserved. DF-1 cells were exposed to different concentrations of purified gp85 for 10 min followed by western blot analysis of kinase activation using specific antibodies. As shown in Fig. 8a, gp85 increased the phosphorylation level of ERK2 and c-Jun, and had no obvious effect on Akt, p38 and JNK activation, which confirmed that ALV activates the ERK2/AP1 pathway via gp85 protein.

We detected a strong phosphorylated ERK2 signal in both the vector control sample and the gag-expressing sample (data not shown) after transfection with cationic lipid transfection reagents, such as Lipofectamine (Invitrogen), which is consistent with another report^27^. Thus, we concluded that the cationic lipid reagents boosted signal transduction^28^, therefore we used electroporation to transiently express gag. Figure 8b shows that electroporation of DF-1 cells with the vector induced visible phosphorylation of ERK2 and c-Jun, however, gag-expressing cells showed a greater degree of ERK2 and c-Jun phosphorylation. In contrast, cells transfected with the control vector or gag-expressing vector did not affect Akt, p38 or JNK activation. This result suggests that gag protein, whose gene is conserved among ALV subgroups, also contributed to the ALV-induced activation of the ERK2/AP1 pathway in DF-1 cells. This is consistent with the findings in Fig. 3c, showing that there was no significant difference between the ALV subgroups in ERK2/AP1 pathway activation.

Lee *et al.*^29^ have reported that phosphatidylinositol-3 (PI-3) kinase is an upstream regulator of MAPK activation in monocyte-derived macrophages (MDM) following HIV-1 gp120 stimulation. In our laboratory, we have previously reported that some exogenous ALVs induced Akt phosphorylation within 15 minutes^30^, while ALV also activated ERK within 15 min at the early infection stage in this study. Therefore, we tested the relationship between PI3K and MAPK activation in response to ALV infection. Consistent with the observations above, CHN06-induced Akt and ERK phosphorylation were PI3K-dependent and MAPKK-dependent, respectively. However, PI3K inhibitor LY294002 had no inhibition effect on ERK activation and MAPKK inhibitor PD98059 could not suppress Akt phosphorylation (Fig. 8c). Taken together, these results suggest that the ALV could induce the activation of the ERK/AP-1 and PI3K/Akt pathways may be in parallel.

### The MAPK pathway regulates viral replication and cellular vascular endothelial growth factor (*VEGF*) mRNA expression

**Figure 9:**
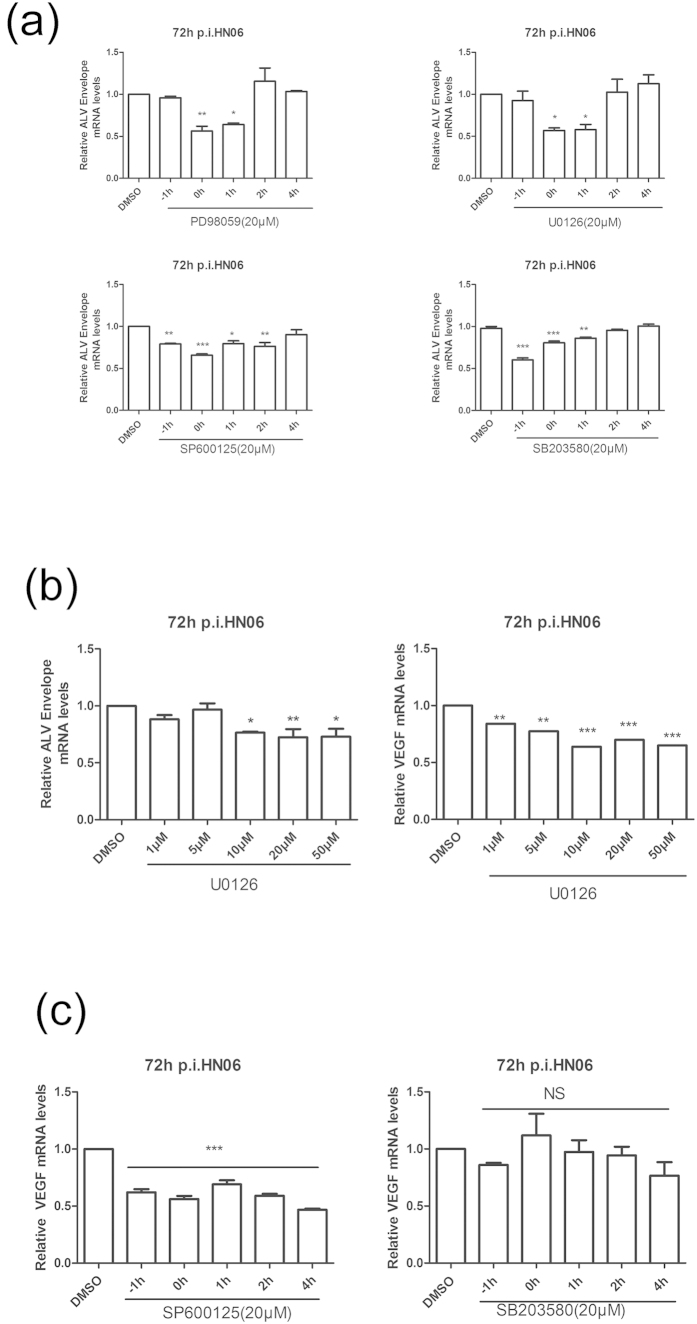
MAPK pathway regulates ALV envelope mRNA and cellular *VEGF* mRNA expression. (**a**) DF-1 cells were treated with PD98059, U0126, SP600125, SB203580 (20 μM) or DMSO (0.1%, v/v) at the indicated times during the infection with CHN06. At 72 h p.i., viral production was monitored by real-time RT-PCR with primers designed for the envelope gene. (**b**) DF-1 cells were simultaneously incubated with CHN06 (10^4^ TCID_50_ 0.2 ml^−1^) and increasing concentrations of U0126 or DMSO (0.1%, v/v). Viral envelope gene and cellular *VEGF* transcription levels were measured by real-time RT-PCR at 72 h p.i. using special primers. (**c**) DF-1 cells were treated with SP600125, SB203580 (20 μM) or DMSO (0.1%, v/v) at the indicated times during the infection with CHN06. At 72 h p.i., the cellular *VEGF* transcription level was monitored by real-time RT-PCR with special primers. Data are representative of two independent experiments, both performed in triplicate. *p < 0.05, **p < 0.01, ***p < 0.001. NS, not significant. Error bars indicate SEM.

Our foregoing results indicated that ALV infection led to MAPK pathway activation. To further detemine the contribution of each MAPK pathway to ALV infection, we investigated the effect of each MAPK pathway inhibitor on viral production measured by viral RNA transcription and viral protein synthesis. As shown in Fig. 9a, addition of PD98059 or U0126 at 0 h p.i. or 1 h p.i. significantly inhibited viral transcription compared with the vehicle-treated control. Similarly, a significant decrease in viral transcription was observed when SP600125 or SB203580 was added at −1, 0 or 1 h p.i. However, no statistical differences in viral transcription were observed when either inhibitor was added at 4 h p.i. Moreover, addition of U0126 at 0 h p.i had an inhibitory effect on the viral RNA levels of ALV-J and on the ALV-J-induced *VEGF* mRNA expression in a dose-dependent manner (Fig. 9b). Treatment of cells with inhibitors at −1, 0, 1, 2 and 4 h p.i. with ALV-J, showed that 20 μM SP600125 treatment resulted in a remarkable decrease in *VEGF* mRNA expression levels, however, incubation with 20 μM SB203580 showed no significant effect (Fig. 9c). These results demonstrated that MAPK primarily acted at an early stage of ALV-J replication cycle. The ERK pathway inhibitor, U0126, and the JNK pathway inhibitor, SP600125, significantly reduced the levels of *VEGF* mRNA expression, whereas the p38 pathway inhibitor, SB203580, exhibited no statistical difference in *VEGF* mRNA expression compared with DMSO treatment before ALV-J infection.

### Effects of MAPK pathway inhibitors on ALV LTR promoter activation

**Figure 10:**
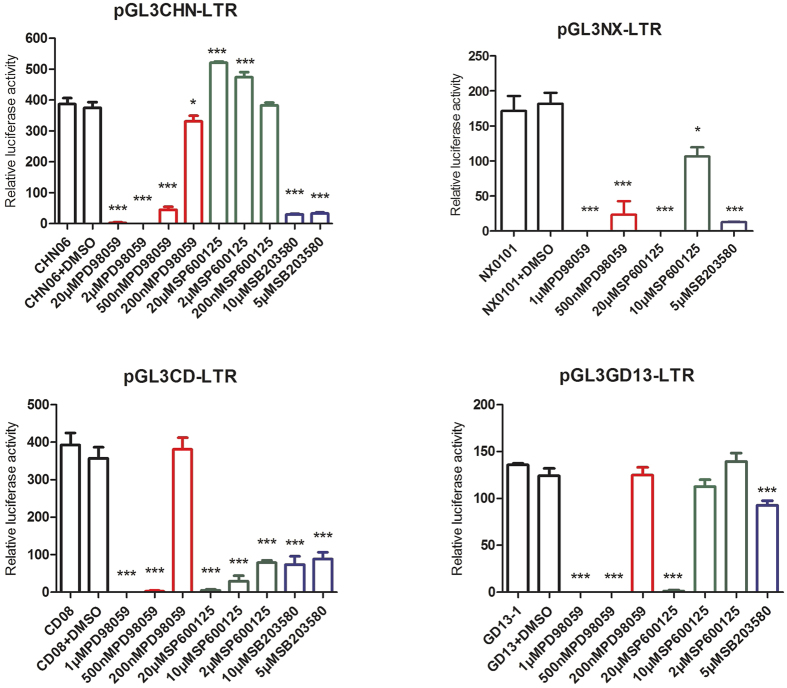
Effects of MAPK pathway inhibitors on ALV LTR promoter activity. DF-1 cells were seeded in 24-well dishes and co-transfected with 0.2 μg pGL3-LTR and 0.02 μg pRL-TK. At 6 h p.i., cells were treated with PD98059 (red bar, 200 nM–20 μM), SP600125 (green bar, 200 nM–20 μM), SB203580 (purple bar, 5 μM–10 μM) or DMSO (second black bar, 0.1%, v/v). At 48 h p.i., cells were lysed for luciferase detection. Data are representative of three independent experiments, performed in triplicate. *p < 0.05, ***p < 0.001. Error bars indicate SEM.

To further investigate the effects of MAPK pathway inhibitors on ALV replication, we studied the effects of the MAPK inhibitors on ALV LTR promoter activity. We found that the relative luciferase activity of the ALV LTR promoter was significantly inhibited by the ERK pathway inhibitor, PD98059, in a dose-dependent manner and with as little as 500 nM. The p38 MAPK pathway inhibitor, SB203580, also significantly inhibited ALV LTR promoter activity, but with a dose of 5 μM, hence, it was far less effective than PD98059. Interestingly, the JNK pathway inhibitor, SP600125 caused a dose-dependent increase in the levels of CHN06 LTR promoter activity and a dose-dependent decrease in the LTR promoter activity levels of NX0101, GD13-1 and CD08 strains (Fig. 10). These results suggest that the MAPK pathways play a role in the activation of the ALV LTR promoter. Moreover, of the three distinct MAPK signaling pathway inhibitors, the ERK pathway inhibitor, PD98059, contributed the most to LTR promoter activity inhibition.

### Effects of MAPK pathway inhibitors on ALV-J protein expression

**Figure 11:**
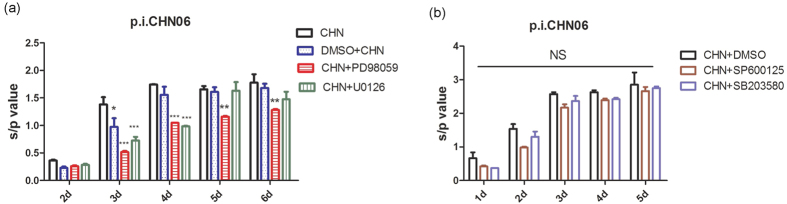
Effects of MAPK pathway inhibitors on ALV-J p27 protein expression. (**a**) DF-1 cells were simultaneously incubated with CHN06 (10^4^ TCID_50_ 0.2 ml^−1^) and PD98059, U0126 (20 μM) or DMSO (0.1%, v/v). (**b**) DF-1 cells were simultaneously incubated with CHN06 (10^4.5^ TCID_50_ 0.2 ml^−1^) and SP600125, SB203580 (20 μM) or DMSO (0.1%, v/v). The inhibitors or DMSO were in the cell culture fluid all the time. ALV-J production was measured by ELISA with anti-p27 antibodies. Data are representative of two independent experiments. *p < 0.05, **p < 0.01, ***p < 0.001. NS, not significant. Error bars indicate SEM.

**Figure 12:**
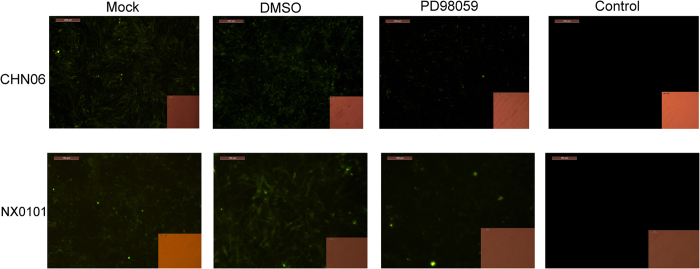
Effects of the ERK pathway inhibitor on ALV-J gp85 protein expression. DF-1 cells were mock treated or simultaneously incubated with 50 μM PD98059 or DMSO (0.1%, v/v) during the infection with CHN06 or NX0101 (10^5^ TCID_50_ 0.2 ml^−1^). At 3 d p.i., monolayers were fixed and stained for gp85 expression, which was visualized by epifluorescent microscopy. Data are representative of two independent experiments. CHN06 group, bar = 200 μm; NX0101 group, bar = 100 μm.

To further validate the ALV inhibition by MAPK pathway inhibitors, we studied the effects of these inhibitors on ALV-J viral protein synthesis. We found that 20 μM PD98059 significantly reduced p27 production of CHN06 strain if compared with the mock and the DMSO control at 3, 4, 5 and 6 days p.i.(DPI), while 20 μM U0126 conspicuously decreased CHN06 strain p27 production at 3 and 4 DPI (Fig. 11a). In contrast, 20 μM SP600125 or 20 μM SB203580 induced no significant CHN06 strain p27 inhibition compared with the DMSO control at different times p.i. (Fig. 11b). Furthermore, 20 μM PD98059 dramatically suppressed gp85 protein expression of ALV-J strains CHN06 and NX0101 (Fig. 12). In contrast, 20 μM SP600125 or 20 μM SB203580 did not affect the gp85 protein expression of ALV-J (data not shown). These results suggest that the ERK/MAPK signaling pathway, but not p38 and JNK signaling pathways, plays an important role in ALV-J viral protein synthesis *in vitro*.

### MAPK is activated in tumor cells

**Figure 13:**
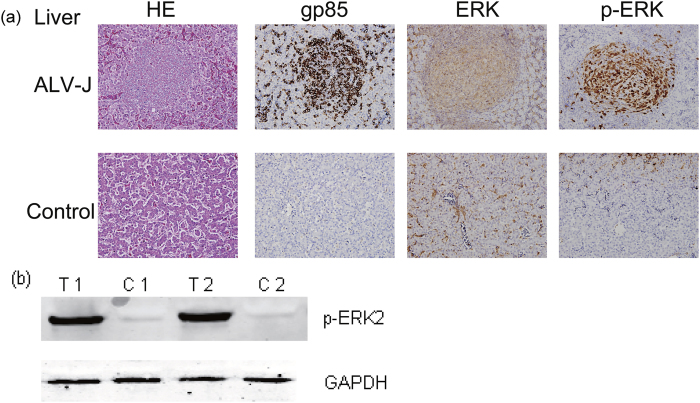
Immunohistochemical and western blot analysis of tumor cells from the liver of ALV-J-infected chickens. (**a**) Fresh tissues were collected and fixed in 10% neutralized buffered formalin, dehydrated, embedded in paraffin wax, and then sliced into 6-μm sections. These sections were routinely stained with hematoxylin and eosin (HE), and examined microscopically. Tissues were further immunohistochemically labeled for gp85, ERK or p-ERK with JE9 monoclonal, anti-p44/42 MAPK or anti-phospho-p44/42 MAPK (Thr202/Tyr204) antibodies, respectively. Bar = 100 μm. (**b**) Western blot analysis detected high expression of p-ERK proteins in myeloid leukosis (ML) samples, but not in the normal samples. Sample identification numbers are indicated above the panel as follows: T, tumor samples; C, non-tumor samples.

Oncogenicity is one of the classical pathologies associated with ALV-J infection^31^. As such, many studies are currently attempting to define the molecular determinants of the ALV-J tumorigenic mechanisms. In light of the numerous reports on the association between the ERK/MAPK pathway and cancer, in addition to our *in vitro* experiments demonstrating the importance of activated ERK2 to ALV replication, we next examined the activation of MAPK in tumor cells from ALV-J-infected chickens. The methods of PCR tests on the genomic DNA of tissues and viral isolation assays have been previously described^32^ and the results were all positive for ALV-J, and negative for ALV-A, ALV-B, REV and MDV. Under the microscope, the tumor cells were observed as relatively uniform large myeloid cells and lymphoid cell hyperplasia in the liver whose morphology are similar to eosinophils (Fig. 13a, HE). Compared with normal cells in the liver, we observed a significant up-regulation of gp85 and pMAPK expression in tumor cells from ALV-J-infected liver. In contrast, only few cells expressing pMAPK were identified in quiescent livers without tumor cells and *gp85* expression (Fig. 13a, p-ERK, *gp85*). Of note, both the tumor cells and normal cells exhibited abundant overall MAPK expression in the liver (Fig. 13a, ERK). These results were also confirmed by western blot analysis of tissue homogenates prepared from tumor tissues and normal tissues (Fig. 13b). These findings suggest that ALV-J-induced tumorigenesis *in vivo* could be correlated with MAPK activation.

## Discussion

Avian retroviruses were originally identified as cancer-inducting filterable agents in chicken neoplasms at the beginning of the 20^th^ century^33^. ALV-J, which originated from a recombination event between ALV and an endogenous retroviral element^34^,^35^, induces a different spectrum of tumors than ALV-A and ALV-B, primarily myeloid leukosis and hemangiomas^3^. Although many studies concentrate on the ALV viral replication and tumorigenesis, the molecular basis of oncogenesis in these tumors is not well understood. In our pervious study, we proposed that ALV provirus has to integrate into the particular genes with a long latency to deregulate specific regulatory pathways and lead to malignant transformation^7^. Exploring cellular signaling pathways involved in virus replication and oncogenesis has become a potential objective. We took advantage of proteomics to identify the differential proteins and intracellular signal transduction pathways in DF-1 infected by ALV-J. The results demonstrated that MAPK1 involved in the ERK/MAPK signaling pathway was up-regulated at 60 h p.i. According to the bioinformatics analysis, Wang *et al.*^36^ have shown that the differentially expressed miRNAs are involved in the tumorigenesis-related MAPK signaling pathway, which may represent a possible signaling pathway involved in the ALV-J-induced tumorigenesis. Hence, we examined the role of the MAPK pathway during exogenous ALV infection and pathogenesis.

The experiments presented in the current study provide evidence that exogenous ALV could induce and sustain activation of ERK2, which is, in turn, required for productive virus replication. The best characterized MAPK module contains ERK1 and ERK2, which is the unique isoform in chickens. ERK2 was found to be phosphorylated at 15 min, 6 h and from 108 h to 132 h post ALV-J infection, suggesting that it was mainly activated at the early and late infection stages. Both ALV-A and ALV-B triggered the production of phosphorylated ERK at 15 min and 120 h p.i. Meanwhile we detected the phosphorylated p38 and c-Jun but not JNK activation at these time points. Besides, ALV-induced c-Jun-phosphorylation could be inhibited by PD98059 instead of SP600125 and SB203580. Hence, it may be active ERK2 that phosphorylates the transcription factor, c-Jun. c-Jun is a member of the AP-1 transcription factor family, and is highly responsive to extracellular signals that control proliferative and apoptotic programs^37^. ERK has been shown to phosphorylate and activate AP-1, which binds to specific genes at binding sites in their promoters^38^,^39^. Dimerization of Jun is required for DNA binding, and DNA binding is necessary for transcriptional activation. All three properties, dimerization, DNA binding and transactivation, are essential for oncogenic transformation^40^. Megan *et al.* have shown that active ERK2 induced the activation of the transcription factors AP-1 and Elk-1, which was sufficient to induce differentiation or transformation depending on the cellular context^41^. *MYC*, *TERT* and *ZIC1* genes are common targets of viral integration and transcriptional deregulation in ALV-J-induced myeloid leukosis^7^. Furthermore, tumor growth and metastasis require angiogenesis, which are regulated by VEGF^20^,^42^,^43^. Wang *et al.*^44^ and Li *et al.*^45^ have found that VEGF-A was overexpressed in DF-1 cells after infection with ALV-J, indicating an increased opportunity for ALV-J to further induce the expression level of VEGF-A to reach the threshold level needed to promote tumorigenesis. AP-1 present in the promoter region of the human *VEGF* gene has been shown to play an important role in the regulation of *VEGF* gene expression^46^,^47^. Intriguingly, 20 μM PD98059 or SP600125 inhibited *VEGF* mRNA expression in ALV-J-infected cells to approximately 50% (*p* < 0.001), whereas SB203580 did not affect it (*p* > 0.05). Actually, compared with the negative control DF-1 cells, the CHN06 strain of ALV-J caused a 1.75-fold up-regulation of *VEGF* mRNA at 72 h p.i. (data not shown). In this regard, both ERK and JNK pathways were involved in the regulation of *VEGF* gene expression after ALV-J infection. Though we did not detect JNK activation at 15min and 120h p.i, ALV infection may activate JNK at other time points. Surprisingly, PD98059 simultaneously inhibited the ALV-induced c-Jun phosphorylation and VEGF-A expression at 120 h p.i. We also found that the mRNA expression of *VEGF* in the tumor tissues was significantly higher than that in the control tissues. In light of our present study, we speculate that the ALV-induced oncogenic transformation was triggered by binding of active c-Jun to the promoters of candidate genes (e.g., *VEGF*, *MYC*, *TERT* and *ZIC1*). In our ongoing study, we hope to identify the promoter sequences and transcription factors’ binding sequences in these chicken candidate genes to further verify the above hypothesis.

There is mounting evidence that activation of MAPK during infection plays an important role in the multiplication of a number of RNA and DNA viruses that replicate in both the nucleus and the cytoplasm, including human immunodeficiency virus type 1 (HIV-1)^48^, influenza virus A and B^49^,^50^, murine coronavirus^51^, Borna disease virus^52^, coxsackievirus B3^53^, BK virus^54^, human cytomegalovirus^55^, herpes simplex virus 2^56^, and the orthopoxvirus vaccinia virus (VCAV)^57^. In this study, we took advantage of four available MAPK signal pathway inhibitors, the ERK pathway inhibitor, PD9859 and U0126, the JNK pathway inhibitor, SP600125, and the p38 pathway inhibitor, SB203580, to further investigate the contribution of each MAPK signaling pathway to ALV virus propagation. To some degree, inhibition of the three kinds of MAPK pathways resulted in a reduction in ALV-J envelope mRNA levels in DF-1 cells, without exception. As ALV transcripts are mainly initiated from the 5’LTR, generating DNA and mRNAs^58^,^59^, we further investigated whether the MAPK inhibitors affect ALV 5’LTR promoter activity. We found that PD98059 and SB203580 substantially reduced the5’LTR promoter activity of ALV subgroups J, A and B in a dose-dependent manner. Specifically, PD98059 almost completely blocked ALV 5’LTR promoter activity with a mere 500nM dose. Of note, SP600125 lead to an obvious increase in the CHN06 5’LTR promoter activity, whereas it inhibited the promoter activity of the NX0101, GD13-1 and CD08 stains. It is conceivable that the differential effect of the JNK inhibitor on the LTR promoter activity was due to the LTR sequence differences among the various strains. The unique 3′ (U3) region of retroviral LTRs has been characterized with transcriptional enhancers and promoters^60^,^61^,^62^,^63^, and multiple *cis*-acting enhancer elements have been verified within the avian retroviruses^64^. Hence, it is possible that MAPK pathways influence LTR promoter activity by interacting with transcription control sequences, further affecting the transcription rate or RNA stability. However, the discrepant effects of SP600125 between CHN06 strain viral RNA level and LTR promoter activity remain unclear. Interestingly, when the ERK pathway was effectively blocked, for example in the presence of U0126 or PD98059, the expression of p27 and gp85 proteins dramatically decreased, whereas SP600125 and SB203580 only slightly decreased the viral protein synthesis. Thus, ERK/MAPK plays active roles in posttranscriptional regulation, supporting the concept that the ERK pathway contributes the most to ALV infection if compared with the other two pathways. This is consistent with other studies on HIV-1 infection^65^.

Previously, we have reported that two kinds of signaling pathway play roles in some exogenous ALVs infection, the PI3K/Akt pathway which is essential for the entry of ALV^30^, and the autophagy pathway inhibited by ALV-J infection^66^. In this study, we first verified that the MAPK pathways are involved in ALV replication and that gp85 or gag protein alone contributes to ERK2/AP-1, but not Akt, p38 and JNK activation. In view of the importance of ERK/MAPK in ALV-J infection, we further explored the relevance of ERK/MAPK activation to ALV-J-associated neoplasm. We showed that the presence of activated ERK2 in ALV-J-induced tumor cells, within which classical targets (gp85 protein) were identified for ALV replication *in vivo*, strongly correlated with virus-induced neoplasm. In other diseases such as visna virus-induced encephalitis^67^ and in human reactive astrocytes in response to CNS injury^68^, chronic activation of ERK1/2 was observed. In this context, the requirement of activated ERK2 for ALV replication is meaningful with regard to our current understanding of cellular proteins and pathways required for avian retroviruses replication and associated oncogenic pathology.

In conclusion, we have shown that infection with exogenous subgroup J, A or B ALVs could activate the ERK/AP1 pathway in associated with viral gp85 and gag proteins, mainly at the early and late infection stages. The ERK/MAPK pathway plays an important role in ALV-J virus replication and associated myeloid leukosis. These findings provide novel insights into the mechanisms of ALV –induced neoplasm.

## Materials and Methods

### Ethics statement

Our animal research was conducted under the guidance of the SCAU’s Institutional Animal Care and Use Committee. The chicken sampling procedures were approved by the Animal Care and Use Committee of Guangdong Province, China.

### Virus propagation and quantification of virus titer

Once DF-1 cells(American Type Culture Collection, Manassas, VA, USA), which are known to be susceptible only to exogenous ALV^69^, had attained 80% confluence, they were infected for 2 h at 37 °C and 5% CO_2_ with the following ALV strains respectively: ALV-J strains CHN06 associated with hemangiomas^70^ and NX0101 associated with myelocytomas^71^, ALV-A strain GD13-1^72^ and ALV-B strain CD08^6^. After the inoculum was removed, maintenance medium containing Dulbecco’s modified Eagle’s medium (DMEM; Gibco, Life Technologies, Carlsbad, CA, USA) with 1% FBS (Gibco) was added and incubated for 6 days. After repeated freezing and thawing three times, the DF-1 cells were centrifuged at 4 °C and 5000 × *g* for 2 min to isolate the supernatants, which were stored as viral stocks at −80 °C until use. The virus titres were determined by serially diluting viral supernatants in DMEM and applying onto DF-1 cells for 6 days in a 96-well plate according to the method described by Reed & Muench^73^, and expressed as TCID_50_ per 0.2 mL. The infected cells were monitored by enzyme-linked immunosorbent assay (ELISA) (ALV Ag Test, IDEXX, Inc., Westbrook, MA) according to the manufacturer’s instructions.

### Cell culture and virus infection

DF-1 cells were seeded in 6-well dishes at 2.0 × 10^6^ cells/ml in DMEM with 10% FBS at 37 °C and 5% CO_2_ until they reached approximately 90% confluence. After starvation in serum-free medium for 6 h, virus stocks diluted in DMEM and DMEM negative control were added to the cells at 37 °C and 5% CO_2_ for 15 min to 168 h. Once the incubation time of the virus-containing media exceeded 4 h, the media was substituted with DMEM containing 0.05% FBS for 6 h to 168 h.

### Sample preparation for proteomic analysis

Sixty hours post-infection (p.i.), mock-infected cells and CHN06-infected cells were washed with phosphate-buffered saline (PBS) and lysed in buffer containing 4% (w/v) 3-[(3-cholamidopropyl)dimethylammonium]-1-propanesulfonate (CHAPS), 7 M urea, 2 M thiourea, 10 mM Tris-HCl and 1 mM EDTA (pH 8.3). The supernatants were collected by centrifugation at 15,000 × *g* for 1 h at 4 °C. Protein concentrations were determined using the Bradford protein assay (Bio-Rad Laboratories, Hercules, CA, USA).

### Concentration of the virus particles by differential centrifugation

As described previously^74^, DF-1 cells infected with ALV-J strain CHN06 for 6 days were centrifuged at 4 °C and 5000 × *g* for 10 min to isolate the DF-1 supernatants after repeated freezing and thawing three times. The supernatants were collected and filtered through a 0.45 μm pore size cellulose acetate filter. A portion of the filtrate was used directly, and the remaining was pelleted by centrifugation for 1 h at 16,000 × *g*. Both of the pellets and the supernatants after centrifugation at 16,000 × *g* were used.

### Plasmid construction

The pGL3-LTR vectors of the different ALV subgroups were constructed by PCR amplification of the long terminal repeat (LTR) region from the genomic DNA of DF-1 cells infected with the ALV strains followed by being cloned into the pGL3-Encher vector (Promega, Madison, WI, USA) using the SmaI and BglII sites. The LTR primers for the CHN06 strain, NX0101 strain and CD08 strain have been previously described^75^. The LTR primers for GD13-1 were 5′-TCCCCCGGGTGTAGTCTTATGCAATACCC-3′ and 5′-GGAAGATCTAATGAAGCCTTCTGCTTCAT-3′. The pGL3-LTR vectors of the different ALV subgroups were sequenced and all had the predicted nucleotide sequences. The pRL-TK plasmid (Promega) was used as a control plasmid that expresses the *Renilla* luciferase reporter gene driven by the herpes simplex virus *thymidine kinase* promoter, and was used to monitor the transfection efficiency.

### The expression of gp85 and gag protein

Recombinant PET28a-gp85 (kindly provided by Dr. Jian zhu Liu, Shandong Agricultural University, China) was expressed in Rosetta (DE3) cells and gp85 protein was purified by a Ni-NTA column and identified by western blot analysis as described previously^76^. pCMV-gag-EGFP was constructed by PCR amplification of the *gag* gene from genomic DNA of GD13-infected DF-1 cells, and subsequently cloned into pCMV-C-EGFP (Beyotime Inst Biotech, Shanghai, China) using the HindIII and SalI sites. The construct was further confirmed by sequencing and transfected into cells by electroporation. Brifely, 10 μg of pCMV-C-EGFP or pCMV-gag-EGFP were electroporated individually into 5.0 × 10^6^ DF-1 cells. After electroporation, cells were maintained in DMEM containing 10% FBS for 24 h, which was substituted with 0.05% DMEM for 48 h.

### Drug cell toxicity test

The protocol for cell toxicity has been reported elsewhere^77^. Cytotoxicity test was determined using Cell Counting Kit-8 dye (Beyotime Inst Biotech) according to the manufacturer’s instructions. Briefly, 5 × 10^3^ DF-1 cells/well were seeded in a 96-well flat-bottomed plate, incubated at 37 °C for 24 h, and then placed in serum-free conditions for another 6 h. Subsequently, cells were treated with drugs at increasing concentrations in the presence of 2% FBS for 48 h. After 10 μL CCK-8 dye was add to each well, cells were incubated at 37 °C for 2 h and the absorbance was determined at 450 nm using a Multiskan FC microplate reader (Thermo Fisher, Shanghai, China).

### Inhibitor treatments and time-course

Phosphoinositide 3-kinase (PI3K)-specific inhibitor LY294002, and the MAPK pathway inhibitors, PD98059, U0126, SB203580 and SP600125, were all obtained from Sigma (St Louis, MO, USA). Treatment of cells with PD98059 (200 nM–50 μM), U0126 (1–50 μM), SB203580 (200 nM–20 μM), SP600125 (200 nM–20 μM), LY294002 (2–20 μM), or solvent (0.1% DMSO, v/v) was performed at the indicated times. For the time-course experiments, PD98059, U0126, SP600125 and SB203580 were added separately to a final concentration of 20 μM at −1, 0, 1, 2 and 4 h p.i. The infection was terminated 72 h p.i. by collecting the monolayers for viral transcription analysis by real-time RT-PCR. To examine the expression level of ALV p27 and env protein, 50 μM PD98059 or DMSO was added to the DF-1 cells for 1 h, which were then infected with CHN06 strain. The cell supernatants were collected for *p27* antigen detection at the indicated times, and the cells were used to examine the env protein by immunofluorescence assay (IFA). All experiments were performed in triplicate.

### Luciferase reporter gene assay

DF-1 cells (~1 × 10^5^) were seeded in 24-well dishes and transfected with pGL3-LTR the following day using Lipofectamine 3000 (Life technologies). To normalize for transfection efficiency, the thymidine kinase (*TK*)-*Renilla* luciferase reporter plasmid (pRL-TK) was added to each transfection. At 6 h post-transfection, cells were treated with the inhibitors of the MAPK signaling pathways. Reporter luciferase activity was measured at 48 h post-transfection using the Dual-Luciferase Reporter Assay System (Promega) and a Tecan Infinite M1000 luminometer (Tecan, Maennedorf, Switzerland) according to the manufacturer’s instructions. Firefly luciferase activity was normalized against the activity of *Renilla* luciferase. Data are representative of three independent experiments, performed in triplicate.

### Western blot analysis

At the indicated times, cell monolayers were washed with PBS and lysed in Cell lysis buffer for Western and IP (Beyotime Inst Biotech). The lysates were collected and incubated on ice for 10 min. Lysates were cleared by centrifugation at 10,000 × *g* for 5 min at 4 °C. The supernatants were analyzed for total protein content with the BCA protein assay kit (Fermentas, Life Technologies). Total protein (20 μg) was resolved by 12% SDS-PAGE and transferred onto nitrocellulose membranes (Whatman, Maidstone, UK). Membranes were blocked with 5% (w/v) skim milk for 1 h at 37 °C, and then incubated overnight at 4 °C with specific rabbit anti-phospho-p44/42 MAPK (Thr202/Tyr204) antibody (Cell Signaling Technology, Danvers, MA, USA), rabbit anti-p44/42 MAPK antibody (Cell Signaling Technology), mouse anti-glyceraldehyde 3-phosphate dehydrogenase (GAPDH) antibody (Beyotime Inst Biotech), rabbit anti-phospho-c-jun (ser73) antibody (Millipore, Billerica, MA, USA), rabbit anti-c-Jun antibody (Abcam), rabbit anti-phospho-JNK (Thr183/Tyr185, Thr221/Tyr223) antibody (Millipore), rabbit anti-JNK1 (EPR17557) antibody (Abcam), rabbit anti-phospho-Akt (Ser473) antibody (Cell Signaling Technology), rabbit anti-Akt antibody (Bioworld Technology, Inc), rabbit anti-phospho-p38 MAPK (Thr180/Tyr182) antibody (Cell Signaling Technology), rabbit anti p38 MAPK antibody (Cell Signaling Technology), rabbit anti-VEGF-A antibody (Abbiotec, USA) or rabbit anti-p27 antibody (kindly provided by Avian Disease and Oncology Laboratory, ADOL). After three rinses with PBS Tween20 (PBST) buffer, the membranes were incubated at 37 °C for 1 h with IRDye 700DX-conjugated anti-rabbit IgG or IRDye 800-conjugated anti-mouse IgG (1:10,000; Rockland Immunochemicals, Limerick, PA, USA) diluted in PBS as the secondary antibody. Membranes were washed three times with PBST, then visualized and analyzed with an Odyssey infrared imaging system (LI-COR Biosciences, Lincoln, NE, USA). The JNK activator, Anisomycin, was purchased from Selleckchem (USA).

### RNA analysis

Total RNA of cells was extracted with RNAfast200 kit (Fastagen, Shanghai, China) followed by cDNA synthesis with the RevertAid First strand cDNA synthesis kit (Fermentas) according to the manufacturer’s instructions. The generated cDNA was then used for real-time PCR amplification. The detailed steps of the SYBR Green I real-time PCR assay for the detection of exogenous ALVs have been previously described^78^. All reactions were performed in triplicate. Finally, real-time quantitative PCR analysis was carried out using the 2^−ΔΔCT^ method^79^.

### ELISA and IFA analysis

At the indicated times p.i., the cell culture supernatants were collected, and the p27 expression level was examined by ELISA following the manufacturer’s instructions. At 72 h p.i. of mock, or ALV-J strains CHN06 or NX0101, DF-1 cells were washed three times with PBS and fixed with 4% paraformaldehyde for 30 min. An ALV-J-specific monoclonal antibody (JE9, kindly provided by Dr. Aijian Qin, Yangzhou University) was used to detect the env protein^80^. Binding of the primary antibodies was detected using FITC-labeled anti-mouse IgG (Sigma) and by fluorescent microscopy (Leica, Solms, Germany). All experiments were performed in triplicate.

### Tissue sample selection and immunohistochemical staining

Suspicious and normal samples were collected from commercial broiler breeder flocks in Guangdong Province, China, in December 2014. Diagnosis of tissue samples was based on characteristic microscopic lesions, molecular analyses and virus isolation. Tissue samples were further diagnosed by immunohistochemical staining with JE9 monoclonal antibody. Anti-p44/42 MAPK antibody was used to immunohistochemically stain tissue sections from ALV-J-positive and uninfected normal chickens. To determine whether pMAPK was expressed in tumor cells, tissues were also immunohistochemically examined for pMAPK expression with anti-phospho-p44/42 MAPK (Thr202/Tyr204) antibody. Binding of the primary antibodies was detected using anti-rabbit-HRP or anti mouse-HRP (Zhongshan Goldenbridge, Beijing, China) and by optical microscopy (Olympus, Tokyo, Japan).

### Statistical analysis

The significance of the differences among the trials was analyzed using the GraphPad Prism version 5.0 software (GraphPad Software, Inc., La Jolla, CA, USA). The results are presented as mean ± SEM, and the statistical significance is represented by *p* values of <0.05, 0.01 or 0.001.

## Additional Information

**How to cite this article**: Dai, M. *et al.* Exogenous avian leukosis virus-induced activation of the ERK/AP1 pathway is required for virus replication and correlates with virus-induced tumorigenesis. *Sci. Rep.* **6**, 19226; doi: 10.1038/srep19226 (2016).
